# Behavioral inhibition in childhood predicts smaller hippocampal volume in adolescent offspring of parents with panic disorder

**DOI:** 10.1038/tp.2015.95

**Published:** 2015-07-21

**Authors:** C E Schwartz, P S Kunwar, D R Hirshfeld-Becker, A Henin, M G Vangel, S L Rauch, J Biederman, J F Rosenbaum

**Affiliations:** 1 Developmental Neuroimaging Laboratory, Psychiatric Neuroscience Program, Department of Psychiatry, Massachusetts General Hospital, Boston, MA, USA; 2 Department of Psychiatry, Harvard Medical School, Boston, MA, USA; 3 Athinoula A. Martinos Center for Biomedical Imaging, Charlestown, MA, USA; 4 Pediatric Psychopharmacology Unit, Department of Psychiatry, Massachusetts General Hospital, Boston, MA, USA; 5 Department of Psychiatry, McLean Hospital, Belmont, MA, USA

## Abstract

Behavioral inhibition (BI) is a genetically influenced behavioral profile seen in 15–20% of 2-year-old children. Children with BI are timid with people, objects and situations that are novel or unfamiliar, and are more reactive physiologically to these challenges as evidenced by higher heart rate, pupillary dilation, vocal cord tension and higher levels of cortisol. BI predisposes to the later development of anxiety, depression and substance abuse. Reduced hippocampal volumes have been observed in anxiety disorders, depression and posttraumatic stress disorder. Animal models have demonstrated that chronic stress can damage the hippocampal formation and implicated cortisol in these effects. We, therefore, hypothesized that the hippocampi of late adolescents who had been behaviorally inhibited as children would be smaller compared with those who had not been inhibited. Hippocampal volume was measured with high-resolution structural magnetic resonance imaging in 43 females and 40 males at 17 years of age who were determined to be BI+ or BI− based on behaviors observed in the laboratory as young children. BI in childhood predicted reduced hippocampal volumes in the adolescents who were offspring of parents with panic disorder, or panic disorder with comorbid major depression. We discuss genetic and environmental factors emanating from both child and parent that may explain these findings. To the best of our knowledge, this is the first study to demonstrate a relationship between the most extensively studied form of temperamentally based human trait anxiety, BI, and hippocampal structure. The reduction in hippocampal volume, as reported by us, suggests a role for the hippocampus in human trait anxiety and anxiety disorder that warrants further investigation.

## Introduction

Behavioral inhibition (BI) is a distinctive, genetically influenced^1, 2^ behavioral profile that is seen in 15–20% of children in the second year of life.^3^ Children born with an inhibited temperament (BI+) are timid with people, objects and situations that are novel or unfamiliar.^3^ More than 25 years before the transdiagnostic and subdiagnostic approaches in RDoC were articulated, a small group of investigators recognized the value of this human phenotype as important orthogonal approach to those then enshrined in the DSM-III for the understanding of psychiatric disorder.^4, 5, 6, 7^ BI was shown to be a risk factor for the subsequent development of anxiety to the unexpected and some anxiety disorders,^5, 7, 8, 9^ as well as depression^6, 10, 11^ and substance abuse^12^ in children, adolescents and adults.

The functional neurocircuitry of BI in humans as a putative intermediate phenotype has become an area of active investigation over the past 10 years. Longitudinal studies have shown that BI observed in childhood, as well as infant high reactivity—a precursor of BI than can be observed at 4 months of age—predict enduring differences in brain function and structure in adults that can be detected after two decades of development.^13, 14, 15, 16, 17, 18, 19^ Although difficult and costly, longitudinal studies remain the gold standard for identifying the developmental trajectory of this phenotype, and require the direct observation of young infants or children and multiple subsequent reassessments. Altered amygdala function in BI subjects, characterized by either increased responses to novel neutral faces and/or sustained responses to neutral faces has been detected in longitudinal samples of subjects classified in infancy or childhood,^13, 17^ a finding replicated in studies of adult subjects who retrospectively reported both inhibited behavior in childhood and current high levels of social inhibition.^20, 21^ Investigators of BI have begun to examine aspects of connectivity between the amygdala and other brain regions.^22, 23, 24^ In addition, other longitudinal cohorts that were first characterized in infancy have demonstrated the involvement of striatal structures in this behavioral profile.^14, 16, 18, 19^

Despite evidence of hippocampal involvement in mood and anxiety disorders, no studies have examined hippocampal structure or function in subjects who were characterized as BI early in life. However, in a study of young adults who respectively identified themselves on questionnaires as both extremely inhibited in childhood and as adults, hippocampal (as well as amygdala) BOLD response failed to habituate to repetitive presentations of neutral faces.^21^

Reduced hippocampal volumes have been observed in depression^25, 26^ and anxiety disorders,^27, 28^ and most frequently and consistently in posttraumatic stress disorder (PTSD).^29, 30, 31, 32, 33, 34^ Given these volumetric findings, and the demonstration that BI in childhood is a prospective risk factor for mood and anxiety disorders later in life,^5, 6, 7, 8, 9, 10, 11^ it is surprising that there are no previous studies of hippocampal structure in BI. Animal models provide clear evidence that severe chronic stress can damage the hippocampal formation.^35^ Cortisol, a stress-related hormone that can be neurotoxic, has been implicated in these effects.^36^ Behaviorally inhibited children are more reactive physiologically as well as behaviorally to unfamiliar or threatening situations, as evidenced by higher heart rate, pupillary dilation, vocal cord tension and, most salient here, higher levels of cortisol at some ages.^3, 37^ We, therefore, hypothesized that the hippocampi of late adolescents who had been behaviorally inhibited early in life would be smaller compared with those classified as not inhibited.

## Materials and methods

The Massachusetts General Hospital institutional review board approved the experimental protocol. Informed consent was obtained after the nature and possible consequences of the study were explained. Hippocampal volumes were examined in 83 late adolescents enrolled in a longitudinal study who had been assessed for BI in the laboratory as young children using standardized batteries as detailed previously.^8, 38, 39^

Each subject underwent two three-dimensional magnetization-prepared rapid gradient-echo (MPRAGE) structural scans on a 3T Siemens (Malvern, PA, USA) TrioTim scanner (128 sagital slices; 1.3 × 1.3 × 1 mm; TR=2530 ms; TE=3.39 ms; flip angle 7°, bandwidth 190 Hz/Px). The two three-dimensional MPRAGE structural scans from each subject were averaged, after motion correction, to create a single high signal-to-noise volume.^40, 41^ This volume was analyzed using Freesurfer v5.0.0 (www.nmr.mgh.harvard.edu/martinos) to calculate left and right hippocampal volumes in cubic millimeters, as previously described.^42, 43^ Freesurfer v5.0.0 computer code is available at https://surfer.nmr.mgh.harvard.edu/fswiki/ReadOnlyCVS. Each scan was manually inspected in simultaneous sagittal, coronal and axial planes by an investigator (PSK) who was masked to the subject's BI status to ensure accurate segmentation. The effects of both BI and familial loading (parental illness type, PIT) on hippocampal volume were analyzed with mixed models (PROC MIXED with LSMEANS/tdiff; SAS v9.3, SAS Institute, Cary, NC, USA), with left and right hippocampal volumes as intra-subject repeated measures, controlling for age, sex, socioeconomic status and intracranial volume. All the statistical tests were two sided. We controlled for socioeconomic status because studies have suggested a relationship between hippocampal volume and social class.^44, 45^ Additional analyses were tested for interactions between sex, BI and PIT.

## Results

High-resolution structural magnetic resonance imaging was used to determine the volume of the left and right hippocampi in 43 females (mean age 17.7±1.9 years) and 40 males (mean age 17.4±1.7 years) from this longitudinal cohort. Twenty-two of the subjects who were imaged had been categorized as behaviorally inhibited (BI+) and 61 as not behaviorally inhibited (BI−) in childhood. Fourteen females and eight males were BI+ 29 females and 32 males were BI−. These subjects were offspring recruited from one of three groups of parents (1) parents with either panic disorder, or panic disorder with comorbid major depression (PD); (2) parents with major depression and no history of panic disorder (pure MD); and (3) control parents without any history of major anxiety disorder or mood disorder (CN).^8, 38, 39^ The young adult subjects therefore had both a measure of familial loading PIT (offspring of PD, pure MD or CN) and a measure of BI (BI+ or BI−). Table 1 gives the mean age and s.d., and the number of subjects in each cell of this 2 × 3 matrix.

Table 2 presents the mean (±s.e.m.) hippocampal volume (average of left and right) for 83 BI+ and BI− subjects in each of the three PIT groups (PD, pure MD, CN). The mean hippocampal volume in BI+ subjects when pooled across the three PIT groups (4367±126 mm^3^) did not differ compared with that in BI− subjects (4330±58 mm^3^; F_(1,71)_=0.09, *P*=0.77). However, those BI+ subjects who were offspring of PD parents did have smaller hippocampal volumes (4080±88 mm^3^) compared with BI− offspring of PD parents (4346±73 mm^3^; *t*_(74)_=2.53, *P*=0.01). This reduction in hippocampal volume was seen only in the BI+ offspring of PD parents, reflecting a significant BI × PIT interaction (F_(2,71)_=3.52, *P*=0.03). In contrast, in the offspring of pure MD parents, no difference was seen in hippocampal volume in BI+ vs BI− subjects (4620±147 mm^3^ vs 4390±99 mm^3^; *t*_(71)_=−1.32, *P*=0.20).

Underlining the fact that familial loading (PIT) alone did not account for the observed differences, neither the hippocampal volume of the BI− offspring of PD parents (4346±73 mm^3^) nor the hippocampal volume of BI− offspring of pure MD parents (4390±99 mm^3^) differed from controls (4254±84 mm^3^). These volumetric differences were seen in both and right hippocampi; there were no significant interactions with side (BI × PIT × sides (F_(2,71)_=0.01, *P*=0.99)). Additional analyses revealed no significant interactions involving sex (BI × sex: F_(1,70)_=0.03, *P*=0.86; PIT × sex: F_(2,70)_=0.3, *P*=0.74; BI × PIT × sex: F_(1,70)_=0.21, *P*=0.65).

## Discussion

We believe this is the first study to demonstrate a relationship between a temperamental profile observed and measured in the laboratory in early childhood, BI and hippocampal structure at adolescence. In this 16-year longitudinal study, a behaviorally inhibited temperament in childhood was associated with smaller bilateral hippocampal volumes in late adolescence in the offspring of parents with PD. The reduction in hippocampal volume was not observed unless both childhood BI and parental PD were present. If we had not possessed these data on parental psychopathology, we would have come to the erroneous conclusion that there was no relationship between BI in childhood and hippocampal volumes in adulthood. Imaging studies of BI have not typically examined such relationships.

What mechanisms and pathways might lead to the decreased hippocampal volume observed in BI+ offspring of parents with PD, and what is the functional relevance of the small hippocampal volumes to state and trait anxiety, including the vulnerability to develop anxiety disorders? We suggest that the reduced hippocampal volume detected in this subset of young adults who had been behaviorally inhibited as young children reflects the interplay of genetic and environmental factors that might emanate from both child and parent. Inhibited children in the second year typically interrupt ongoing play, cease vocalizing, seek comfort from a familiar person or withdraw when presented with people, objects and situations that are novel or unfamiliar. When presented with such situations in the laboratory, behaviorally inhibited children show enhanced physiological reactivity as evidenced by a higher heart rate, decreased heart rate variability, pupillary dilation, increased vocal cord tension and higher levels of cortisol.^3, 37^ The hippocampus is densely populated with receptors for cortisol; stress and glucocorticoids not only cause cell and atrophy but also inhibit adult neurogenesis.^46^ Animal studies suggest that impairing neurogenesis in the hippocampi of adults slows the recovery of glucocorticoid levels after stress responses and increases depression-like behaviors in behavioral tests commonly used to assess antidepressant response.^47^ Because stress and glucocorticoids regulate the production of new neurons, a positively reinforcing toxic loop could be created for aberrant and pathological responses to stress in the future. Such a mechanism of reduced hippocampal reserve might be important in the genesis and subsequent maintenance of clinical anxiety and mood disorder in humans.

It has been suggested that an impairment in contextual fear discrimination could cause a bias towards encoding potentially ambiguous cues as threatening (the thud of fireworks vs the thud of a mortar explosion), providing a possible explanation for the overgeneralization seen in PTSD and PD.^48, 49^ Neuroimaging studies have implicated the hippocampus in the contextual modulation of both fear-conditioning^50^ and fear-extinction recall^51^ in healthy adults. Deficient extinction retention and attendant decreases in hippocampal activation have been reported in patients with PTSD when compared with subjects without PTSD who have been exposed to major emotional trauma.^52^ Antidepressant medications, which are also anxiolytic, increase adult hippocampal neurogenesis.^53, 54, 55^ A genetically induced increase in hippocampal neurogenesis enhances the ability of animals to differentiate between two similar conditioning contexts.^56^ Small hippocampal volumes, on the other hand, are associated with a failure to learn to discriminate between conditioned contexts in a contextual fear-conditioning paradigm in humans.^57^ A study of autonomic responses and contingency awareness during fear conditioning demonstrated that individuals with smaller hippocampal volumes were less successful in identifying the safety signal represented by the conditioned stimulus that was never followed by a painful electric shock during acquisition.^58^ Another line of research has demonstrated reduced hippocampal volume in subjects with childhood maltreatment^31, 32, 34, 45^ and one longitudinal study discovered that a reduction in hippocampal volumes not only mediated the relationship between early-life stress and trait anxiety but also predicted the levels of anxiety symptoms in response to stress 1 year later.^59^

The studies reviewed above suggest a functional link between the small hippocampal volumes in BI+ offspring of parents with PD, and the increased vulnerability of behavioral inhibited subjects to anxiety and anxiety disorders. Larger samples would permit mediation analyses to explore the pathways between parental illness, BI, hippocampal volume and specific illnesses or symptoms in the offspring. However, consistent with the notion of antecedent-reduced hippocampal reserve, a twin study has suggested that a smaller hippocampal volume may be a pre-existing vulnerability factor for the development of PTSD.^60^ Unfortunately, the incidence of serious trauma and PTSD is too low in our sample to directly investigate this potential link.

Parental PD, the second factor required for the observed reduction in hippocampal volume, could relate to the influence of parenting style and family stress on the developmental trajectory of children with BI.^61, 62, 63, 64, 65, 66, 67, 68^ A particularly elegant longitudinal study by Kiel and Buss^68^ measured both BI and maternal overprotective behavior at 2 years, as predictors of social withdrawal at 5 years of age when the children entered kindergarten. The authors defined overprotective behavior as protective behavior that occurs in novel or uncertain situations that, despite inducing discomfort for some children, present no actual danger.^68^ Path analyses suggested that at 2 years of age, temperamentally fearful children elicited protective behaviors from their mothers (occurring most strongly when mothers accurately predicted that their child would become upset by an upcoming situation), and that this overprotective behavior at 2 years of age, in turn, predicted social withdrawal 3 years later.^68^ Taking the next step along this translational path, investigators have sought to influence the interactions of parents with their BI+ offspring to decrease the probability of developing anxiety disorders.^69, 70, 71, 72, 73^ One longitudinal intervention study targeted the parents of BI+ children between 3 and 5 years of age at risk for anxiety with a brief education program that specifically addressed the role of overprotective behavior in maintaining anxiety. One, 2 and 3 years after the parental intervention,^70, 73^ the offspring of parents in the intervention group had lower rates of anxiety disorders—and 11 years later, at age 15, they showed lower rates of both anxiety and depression, when compared with a passively-monitored control group.^69^ These studies of parenting and studies of interventions aimed at changing parental behaviors support the plausibility and developmental salience of the BI × PIT interaction reported here and, in turn, generate testable hypotheses regarding mechanistic paths to the reduced hippocampal volumes detected in BI+ offspring of parents with PD. For example, if parents with PD were more likely to interact with their inhibited children in a stress-inducing overprotective manner that led to elevated cortisol levels in their child, this could impair hippocampal neurogenesis resulting in the decreased volumes that we detected.

Turning to consider genetic factors, twin studies have demonstrated the substantial influence of genes on inhibited temperament. In a population-based sample of more than 4500 4-year-old twin pairs,^74^ the largest heritability estimates (64–76%) were observed for inhibition/shyness. Overall, estimates of the heritability of BI in these studies have ranged from 0.41–0.76, with estimates as high as >0.9 for extreme BI.^1, 74, 75, 76, 77, 78^ These estimates are substantially higher than those reported for social anxiety disorder and other anxiety disorders (range 20–40%), suggesting that the temperamental precursor phenotype is under stronger genetic influence than the disorders to which it predisposes. Therefore, although our discussion to this point has posited a central role for environmental stress in generating the decreased hippocampal volumes in the subset of behaviorally inhibited adolescents reported here, it is also possible that the reduction in hippocampal volumes reported could have genetic determinants, and constitute a pre-existing risk factor that is subsequently amplified by experience during development. In line with this possibility, there is evidence that genetic factors independent of environmental stress can influence hippocampal size. A longitudinal study in monkeys found that hippocampal volumes in early adulthood did not differ on the basis of experimentally induced stress in the postnatal period, but the observed variation in monkey hippocampal volumes was heritable.^79^

Given that the reduction in hippocampal volume in our study occurred only in the subset of those BI+ subjects with PD parents, BI and PD might be characterized by partially overlapping polygenetic influences, both of which are required for the observed change in hippocampal structure. Consistent with this notion, and in light of the role, as suggested by us, of cortisol in these findings, it is interesting that an association between BI and a microsatellite marker tightly linked to the corticotropin-releasing hormone gene in 84 families of children assessed for BI was particularly marked in the offspring of parents with PD.^80^ This marker and multiple single-nucleotide polymorphisms encompassing the corticotropin-releasing hormone gene were subsequently genotyped in an expanded sample of families of children at risk for PD.^81^ The BI phenotype remained significantly associated with the microsatellite marker and was associated with several single-nucleotide polymorphisms including a single-nucleotide polymorphism in the coding sequence of the corticotropin-releasing hormone gene; haplotype-specific tests revealed an association for a haplotype comprising all the markers.^81^

### Methodological considerations, limitations and directions for future inquiry

The present study cannot definitively disentangle the potential contributions of the genetic, developmental and environmental mechanisms to the behaviorally inhibited phenotype, including the structural differences reported here. New longitudinal studies will be needed to identify and image high-reactive infants, the infant precursor profile to BI in childhood and define the state of brain circuitry at the very beginning of the developmental trajectory of this phenotype, while gathering home-based measures of parenting and other environmental variables such as social class and the size of social networks, in concert with genome-wide association studies and studies of gene regulation and expression. Following such a cohort of infants throughout their development into young adults would elucidate both causality and mechanism, and suggest new strategies for early intervention. As the above discussion of the present findings demonstrates, the study of BI has required translational bridges between psychology and psychiatry, with a longitudinal multimethods developmental approach that could be a fruitful model for the study of other psychiatric symptomatology such as psychosis.

This report demonstrates that data about parental psychopathology may reveal relationships between brain circuitry and BI in their children that are otherwise not detectable. The finding that hippocampal volume in the offspring of pure MD parents did not differ significantly on the basis of BI status must be interpreted with caution, given the small number of subjects in the BI+/pure MD cell relative to the other main cells of interest. Although this represents a limitation of this study, one hint that this cell might convey a meaningful biological signal in the opposite direction of that seen in the offspring of PD parents was the fact that the mean hippocampal volume of subjects in this cell (4660±138 mm^3^) was larger than the hippocampal volume of the BI− offspring of controls (4262±67 mm^3^; *t*_(74)_=−2.60, *P*=0.01). Studies in larger samples will be required to clarify this. The fact that there was just one behaviorally inhibited subject among the offspring of the control parents (that is, families without parental PD or major depression) is consistent with reports in larger samples that BI is rare in parents without a history of PD or major depression.^4, 38^

The preponderance of neuroimaging literature on anxiety and anxiety disorders in humans to date has focused on the amygdala, ventromedial prefrontal cortex and anterior cingulate. The reduction in hippocampal volume reported in this longitudinal study in a subset of behaviorally inhibited adolescents, when considered in concert with a previous functional magnetic resonance imaging study showing impaired hippocampal habituation in adults who retrospectively identified themselves as inhibited children^21^ and recent studies in non-human primate models of anxious temperament,^82, 83, 84, 85^ suggests that the hippocampus has an important role in human trait anxiety and anxiety disorder that warrants further investigation.

## Figures and Tables

**Table 1:** Age (mean years±s.d.) and number of subjects (*n*) by childhood behavioral inhibition and parental illness type

*Childhood behavioral inhibition*	*Parental illness type (PIT)*			*All*
	*PD*	*Pure MD*	*Controls*	
BI−	17.68±2.04 (*n*=29)	17.71±1.28 (*n*=11)	17.24±1.86 (*n*=21)	17.54±1.85 (*n*=61)
BI+	17.46±1.76 (*n*=16)	18.08±2.39 (*n*=5)	18.36 (*n*=1)	17.64±1.84 (*n*=22)

Abbreviations: BI, behavioral inhibition; MD, major depression; PD, panic disorder.

**Table 2:** Hippocampal volume (mean R/L mm^3^±s.e.m.) at adolescence (*n*=83) by childhood behavioral inhibition and parental illness type

*Childhood behavioral inhibition*	*Parental illness type (PIT)*			*All*
	*PD*	*Pure MD*	*Controls*	
BI−	4346±73	4390±99	4254±84	4330±58
BI+	4080±88	4620±147	4401±332^a^	4367±126

Abbreviations: BI, behavioral inhibition; MD, major depression; PD, panic disorder.

a *n*=1 for this cell (Table 1).
